# Tumor DNA from tumor *in situ* fluid was used to track evolution of glioma under first-line treatment

**DOI:** 10.3389/fonc.2025.1581173

**Published:** 2025-10-16

**Authors:** Jinliang Yu, Yushuai Gao, Jiubing Zhang, Dayang Wang, Kaiyuan Deng, Shuang Wu, Ziyue Zhang, Zhaoyue Yan, Guanzheng Liu, Liujian Dong, Tao Li, Shubin Feng, Xingyao Bu

**Affiliations:** ^1^ Department of Neurosurgery, Children’s Hospital Affiliated to Zhengzhou University, Henan Children’s Hospital, Zhengzhou Children’s Hospital, Zhengzhou, China; ^2^ Department of Neurosurgery, Henan Provincial People’s Hospital, Zhengzhou University People’s Hospital, Henan University People’s Hospital, Zhengzhou, China

**Keywords:** pseudoprogression, glioblastoma, early recurrence, ctDNA relapse, tumor *in situ* fluid

## Abstract

**Introduction:**

Few studies have tracked the genetic evolution of glioma recurrence in patients after surgery. We conducted a systematic review through an innovative sampling method, which made post-operative follow-up possible. Tumor DNA from Tumor in situ fluid (TISF) was used to trace the genetic landscape of gliomas at different stages of recurrence and evolution.

**Methods:**

We recruited 60 patients with WHOII-III gliomas diagnosed more than 6 years ago. We performed whole exome sequencing (WES) of primary tumor tissues and paired TISF to identify somatic mutations by personalized, tumor-informative TISF-DNA testing. TISF and recurrent tumor tissues were collected at simultaneous and 2-3 month routine visits. Patients were followed up for clinical recurrence.

**Results:**

In gliomas dominated by genomic alleles with low frequency (variant allele fraction, VAF < 1%), imaging residues had higher VAF (p = 0.016), and patients with postoperative recurrence also had higher VAF (p < 0.0001). Under the pressure of treatment, multiple mutations gradually increased with tumor evolution, and dominant high-frequency mutation gradually appeared. Samples of relapsed TISF contained much more abundant clonal mutations. Sequencing of relapsed tumor tissue and relapsed TISF samples showed high consistency in mutation detection and estimation of allele frequency (p < 0.0001, VAF correlation, R2 = 0.8737). In patients under continuous surveillance, the tumors at different stages showed heterogeneity. We determined that TISF may detect elevated Tumor DNA VAF prior to positive imaging findings and effectively identify patients with pseudoprogression.

**Discussion:**

TISF-DNA showed high consistency with tumor tissue, showing the genetic landscape of glioma at different stages after surgery. It can even be used to identify false progression during glioma treatment. Even in the absence of imaging findings, glioma DNA recurrence should arouse clinical concern and induce new research.

## Introduction

Glioma is the most common primary intracranial malignancy, and there is no standard treatment for recurrence (1). Recent advances have shown significant epigenetic and environmental heterogeneity within gliomas, which are linked together to lead to extreme phenotypic heterogeneity at the cellular level, providing multiple therapeutic resistance mechanisms and complicating the treatment of gliomas for recurrence (2).

Enhanced detection of minimal residual disease by targeted sequencing of phased variants in circulating tumor DNA (3), guiding adjuvant immunotherapy (4), tumor early relapse detection (5), tracking the evolutionary dynamics and heterogeneity of tumors (6), Positive ctDNA is considered as possible evidence of early cancer molecular residual disease (MRD) (7–10). The clinical correlation between ctDNA and cancer is the direction of future clinical decision-making. Recent studies evaluating ctDNA in different tumor types, including gliomas, have shown that ctDNA and genetic profiles from tumor or metastatic biopsy tissue samples have the same ability to track changes in mutations and mutation patterns (11, 12). However, there are currently limited studies on gliomas. Recent studies have shown that circulating tumor DNA from the cerebrospinal fluid is more representative than circulating tumor DNA from blood (13). However, CSF-ctDNA is positive only when tumor load is high (14–16), adjacent to cerebrospinal fluid, tumor progression, and glioma genomic evolution information lost under therapeutic pressure. TISF-DNA as a biomarker of glioma load is our latest discovery (17). It overcomes the shortcomings of tissue-based biomarkers and enables rapid sampling and multiple continuous monitoring to detect tumor heterogeneity on time. TISF-DNA was more sensitive to low tumor load. At the same time, the TISF collection process was even less invasive than lumbar puncture, and TISF was superior to cerebrospinal fluid (CSF) even when gliomas were adjacent to CSF systems (18). We found a higher positive rate of TISF-DNA in the early postoperative period, and a higher concentration of cfDNA than CSF, even in paired samples tested at the same time (19). However, continuous monitoring is needed to understand the evolution of postoperative glioma recurrence, and relevant studies are still insufficient at present.

We conducted an observational study to monitor mutated genes in gliomas at different stages of first-line treatment using continuous TISF-DNA testing and investigate its potential relevance to clinical outcomes.

## Materials and methods

### Patients and sample collection

**Figure 1:**
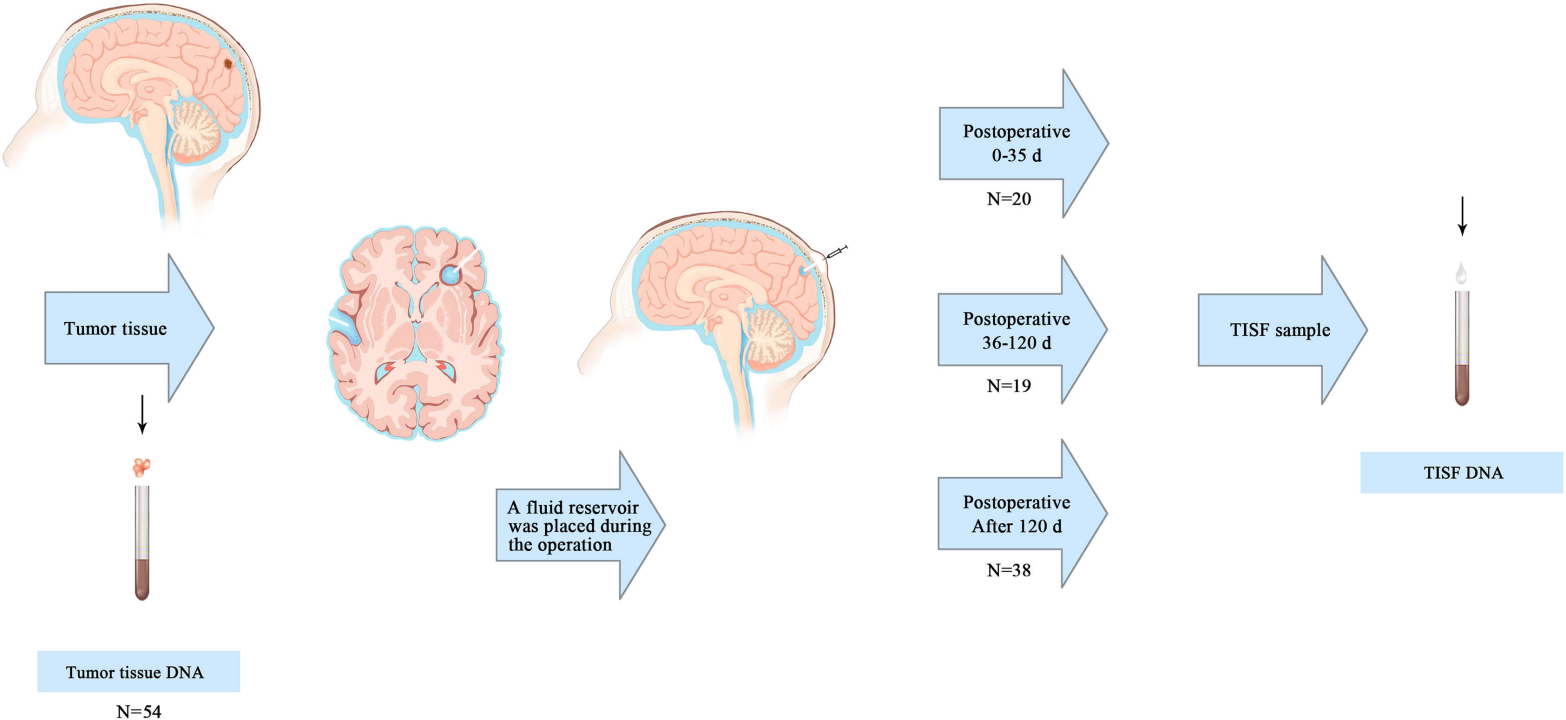
Primary tumor and TISF samples were obtained. The primary tumor samples were obtained from surgically removed tumor tissue. A reservoir sac with a catheter is placed after the primary tumor is removed to form a residual cavity. The catheter ends in the tumor cavity, and the reservoir sac is placed under the scalp. TISF samples were obtained at three postoperative time points: 0-35d, 36-120d, and after120d.

This prospective observational study was conducted on January 1, 2016, at the People’s Hospital of Henan Province on January 31, 2022. The primary tumor was resected and a fluid reservoir was placed in the residual cavity after tumor resection for postoperative TISF collection (17) (**Figure 1**). The primary tumor samples were obtained by surgery, and the NCCN regimen was used to guide the treatment after surgery. TISF samples were collected at different postoperative times: 0-35d, 36-120d, and more than 120d after surgery (tumor progression according to RANO standard, T1 enhancement increased by ≥ 25%, T2/FLAIR increased, and new lesions and clinical manifestations deteriorated). Tumor volumes of all patients were determined before and after surgery by manual segmentation of corresponding MRI images using a 3D slicer (version 5.0.2). Contrast-enhanced T1WI and T2WI/T2-flair images were the primary references for determining tumor boundaries. During patchy enhancement, the high-intensity signal on T2WI/T2Flair is used to delineate tumor boundaries. Total resection rate of tumor = (preoperative tumor volume - postoperative tumor volume)/preoperative tumor volume. A removal rate less than 90% is considered residue. This process is done by a single radiologist (**Supplementary Table S1**). Five patients underwent a second surgical resection of the tumor after radiographic recurrence was found. Fresh tumor tissue comes from surgical resection, and HE staining specimens contain more than 70% of tumor cells, which neuropathologists have confirmed. In addition, matching blood samples from each patient were taken to filter for mutations from normal DNA (**Supplementary Table S2**). A brain tumor gene panel was designed to screen for tumor mutations (**Supplementary Table S3**). Grade III-IV glioma patients were followed up every 4-6 weeks, and grade II glioma patients were followed up every 2-3 months. All patients underwent MRI at each follow-up evaluation.

### Targeted sequencing analysis of tumor-associated DNA

All clinical TISF samples and tumor tissue samples were detected by Next-generation sequencing. QIAamp DNA Tissue and Blood Kit for Genomic DNA (Qiagen; Germantown, MD, USA) extract. TISF sample and blood sample were centrifuged in an EDTA tube at 1900 g for 10 min, and the precipitate particles were frozen at −80°C. The supernatant was centrifuged at 16000 g for 10 min and transferred to −80°C for preservation. CfDNA was extracted from TISF and blood supernatant using a Mag-MAX CellFree DNA isolation kit (Thermo Fisher Scientific, Waltham, MA, USA). Finally, all segregated DNA was quantified using the Qubit 2.0 Fluorometer with the Qubit dsDNA HS Assay kit (Life Technologies; Carlsbad, CA, USA).

The isolated DNA was cut into 150-200 bp fragments using Covaris M220 Focused-ultrasonicator™ Instrument (Covaris; Woburn, MA, USA). Following the manufacturer’s direction9,10 to construct Fragmented DNA and ctDNA libraries with the KAPA HTP Library Preparation Kit (Illumina platforms; KAPA Biosystems; Wilmington, MA, USA). The DNA sequencing was based on a novaseq high-throughput sequencing platform. After sequencing, we adopted such criteria that a mutation had an allele fraction of ≥ 0.1%, and a total of ≥ 4 reads were considered existing in liquid samples. Known recurrent loci were further manually checked with Integrative Genomics Viewer (IGV v2.3.34) in the target sample comparing to the normal blood DNA. Using the dbNSFP and the Exome Aggregation Consortium (ExAC) database to exclude either benign mutations with pp2_hdiv score < 0.452 or polymorphic nonsynonymous mutations sites. In the end, all detected mutations were annotated for genes using ANNOVAR, Oncotator and Vep.

### Statistical analysis

Graph drawing was completed in GraphPad Prism(Version 8.0C), heat maps were generated by TBtools, and statistical tests were performed by SPSS (Version 23.0; Armonk, NY, IBM Corp), Fisher’s exact test for categorical variables, Wilcoxon test, and Mann-Whitney(rank-sum) test or Kruskal-Wallis test for continuous variables.

## Results

### Patient baseline characteristics

We isolated TISF-DNA from 60 patients with primary glioma of the brain central nervous system. (**Supplementary Table S1**). The mean age was 51.5 years (range 21-78 years), and the tumor grade was WHO grade II-IV. Most of the tumors were located in the frontal lobe (n = 19) and temporal lobe (n = 10), temporoparietal lobe (n = 8), cerebellum (n = 2), thalamus (n = 1), and some in both lobes (n = 12), while the remaining tumors were located in the parietal lobe (n = 8). The genome of the primary tumor (n = 54) represents the characteristics of glioma before surgery. 0-35d after surgery (n = 20) represents the genomic characteristics of minimal residual disease in the tumor lumen early after surgical resection. 36-120d after surgery (n = 19) represents the genomic characteristics of glioma undergoing chemotherapy (low grade) and chemoradiotherapy (high grade). 120d after surgery (n = 38) represents genomic characteristics of further chemotherapy during treatment. Radiographic tumor recurrence was found in 26 patients at the time of sampling.

### Genomic landscapes of glioma at various stages under first-line treatment

**Figure 2:**
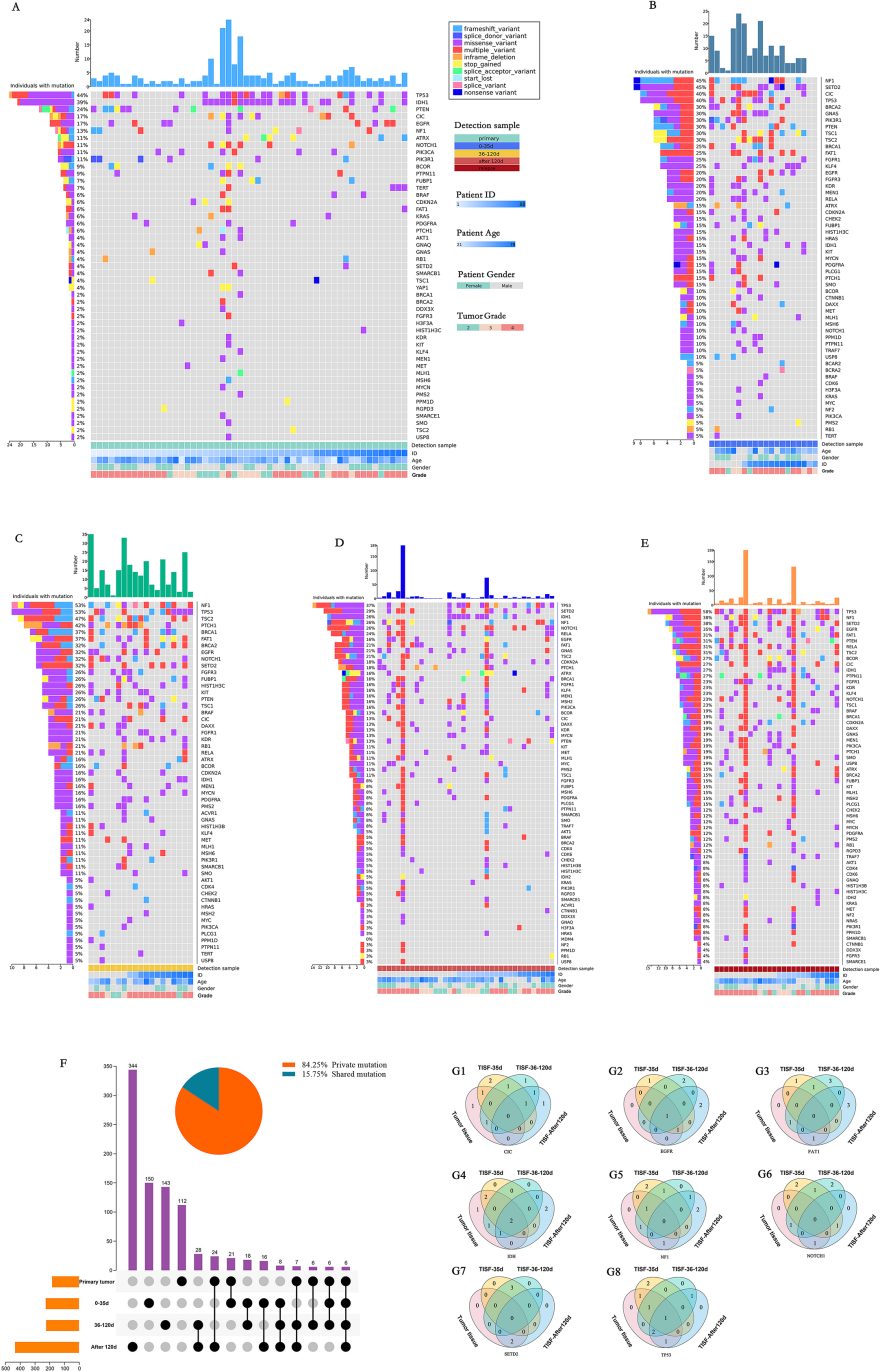
Mutational characteristics for alterations detected by deep sequencing of the TISF-DNA and tumor tissue DNA. **(A-E)** They were primary tumor tissue samples, TISF samples within 0-35 days after surgery, TISF samples 36-120 days after surgery, TISF samples 120 days after surgery, and TISF samples of recurrent tumors, including missense variant, Inframe deletion, and deletion. Frameshift variant, splice acceptor variant, stop gained, Multiple variant, frameshift variant, splice donor variant, start lost, splice variant, nonsense variant. **(F)** Shared and private mutation characteristics in glioma map loci at different stages. **(G1-G8)** TISF samples were obtained from 14 consecutive patients. TISF samples were obtained from 14 consecutive patients. The number of patients with high frequency altered genes CIC, EGFR, FAT1, IDH, NF1, NOTCH1, SETD2, and TP53 in each test was shown.

In general, the altered genes are different at different stages of the treatment process, but there is some similarity. For the primary tumor (**Figure 2A**), The most frequently altered genes were TP53 (44%), IDH1 (39%), PTEN (24%), CIC (17%), EGFR (17%), NF1 (13%). At the stage of 0-35d after surgery (**Figure 2B**), The most frequently altered genes were NF1 (45%), SETD2 (45%), CIC (40%), TP53 (40%), BRCA2 (30%), GNAS (30%). After treatment 36-120 days after surgery (**Figure 2C**), The most frequently altered genes were NF1 (53%), TP53 (53%), TSC2 (47%), PTCH1 (42%), BRCA1 (37%), FAT1 (37%). 120d after surgery (**Figure 2D**), The most frequently altered genes were TP53 (37%), SETD2 (39%), IDH1 (26%), NF1 (26%), NOTCH1 (26%), RELA (24%). For radiographic recurrence (**Figure 2E**), the mutation rates were TP53 (58%), NF1 (38%), SETD2 (38%), EGFR (35%), FAT1 (31%), PTEN (31%). Interestingly, during the whole treatment process, TP53 and NF1 continued to have high frequently altered, and EGFR, PTEN and SETD2 also had high frequently altered. They may be the driving mutations retained when tumors recur. Gliomas at different stages were highly heterogeneous during postoperative treatment, with only 15.75% shared mutation rate and 84.25% private mutation rate (**Figure 2F**). Fourteen patients were sampled and monitored continuously. Among the genes with high mutation frequency, only EGFR (n = 1, 14.3%), IDH (n = 2, 22.2%) and TP53 (n = 1, 11.1%) were present in all monitored samples (**Figures 2G1-G8**). Mutations present in primary tumors were also found in TISF-DNA, such as CIC (n = 2, 20.0%), FAT1 (n = 2, 20.0%), NF1 (n = 3, 33.3%), NOTCH1 (n = 2, 25.0%) and SETD2 (n = 2, 33.3%). However, more interestingly, mutations were found in TISF but not in the primary tumor in many patients, such as CIC (n = 7, 70.0%), EGFR (n = 5, 71.4%), FAT1 (n = 8, 80.0%), IDH (n = 2, 22.2%), NF1 (n = 6, 66.7%), NOTCH1 (n = 6, 75.0%), SETD2 (n = 4, 66.7%), TP53 (n = 3, 33.3%).

### Genomic VAF changes under first-line treatment

**Figure 3:**
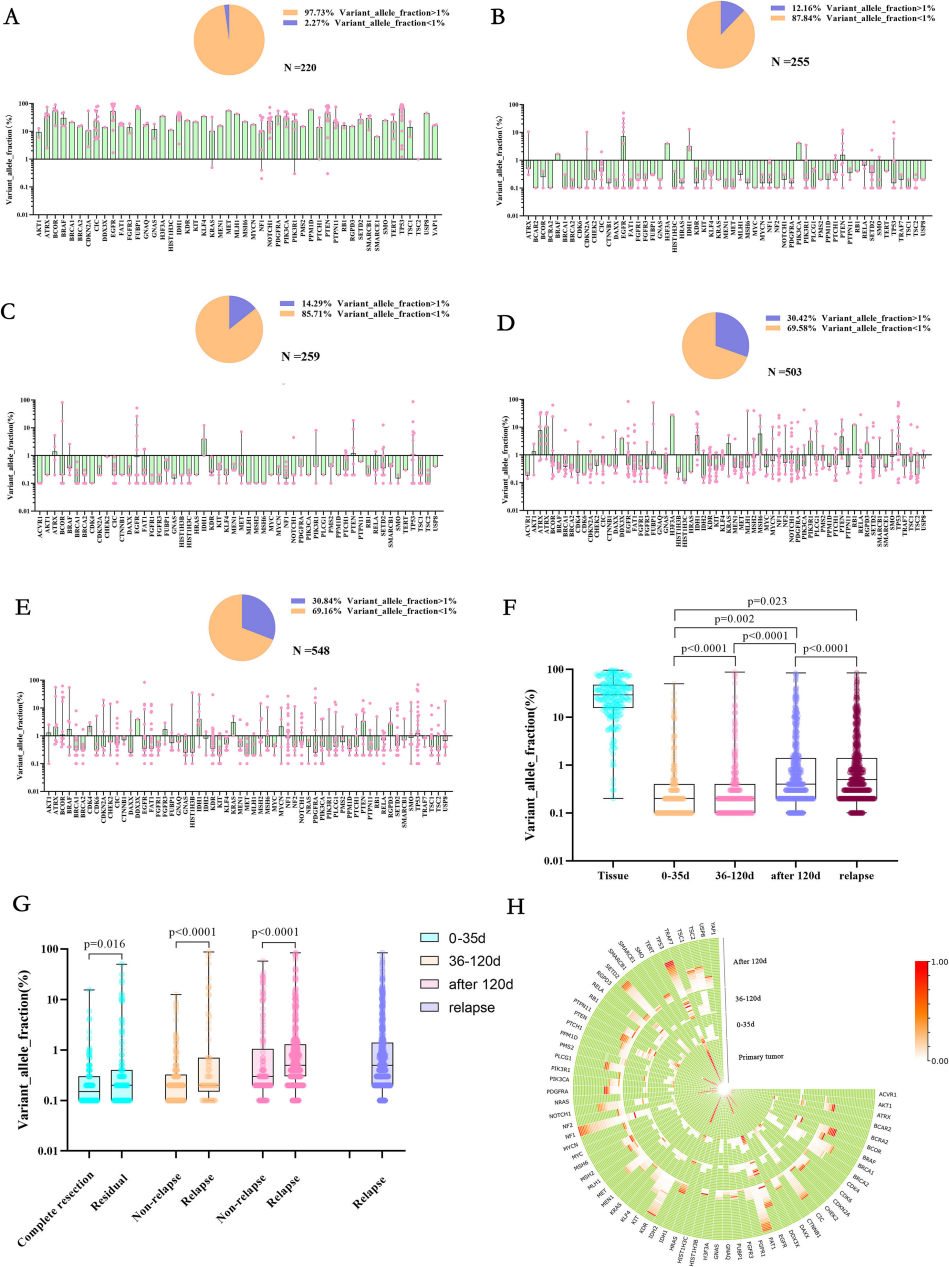
Allele variation frequency in the genome at different stages of first-line treatment for glioma. **(A-E)** They were primary tumor tissue samples, TISF samples within 0-35 days after surgery, TISF samples 36-120 days after surgery, TISF samples 120 days after surgery, and TISF samples of recurrent tumors, VAF distribution of different mutated genes, and low-frequency mutation (VAF<1%) ratio shows. **(F)** Allele Variation frequency of genes in all periods is integrated. High heat represents high VAF; the closer it is to the periphery, the closer it is to the recurrence time, and the center represents the primary tumor sample. **(G)** Allele Variation frequency of mutated genes in different periods increased with tumor evolution, showing significant statistical differences. **(H)** Allele Variation frequency in TISF samples of patients with postoperative imaging residue compared with patients with complete imaging resection and patients with recurrence compared with patients without recurrence was significantly different.

We found that the primary tumor tissue had such a high VAF (**Figure 3A**) that 97.37% of the mutation had a VAF value greater than 1%, and only 2.27% of the low-frequency mutation. However, in the early stage after tumor resection (within 35d), 87.84% of the low-frequency mutation had a VAF value of less than 1% (**Figure 3B**). Furthermore, only 12.16% of the mutation with a VAF value greater than 1%. With further postoperative treatment (36-120 days), mutations with VAF greater than 1% increased slightly (**Figure 3C**) to 14.29% 120 days after surgery, and mutations with VAF greater than 1% were significantly increased, accounting for 30.42% (**Figure 3D**). In TISF samples from patients with recurrence, mutations with VAF greater than 1% were found to be slightly higher overall, accounting for 30.84% (**Figures 3E, G**, p < 0.0001). In addition, we also found that patients with higher VAF within 35 days after surgery may be associated with postoperative residual. Imaging showed that patients with significant residual VAF were more extensive than those with complete radiographic resection (**Figure 3H**, p = 0.016). In contrast, patients with radiographic recurrence during postoperative treatment had higher VAF than those without recurrence (**Figure 3H**, p < 0.0001, p < 0.0001).

### Changes in specific gene types in glioma under first-line treatment

**Figure 4:**
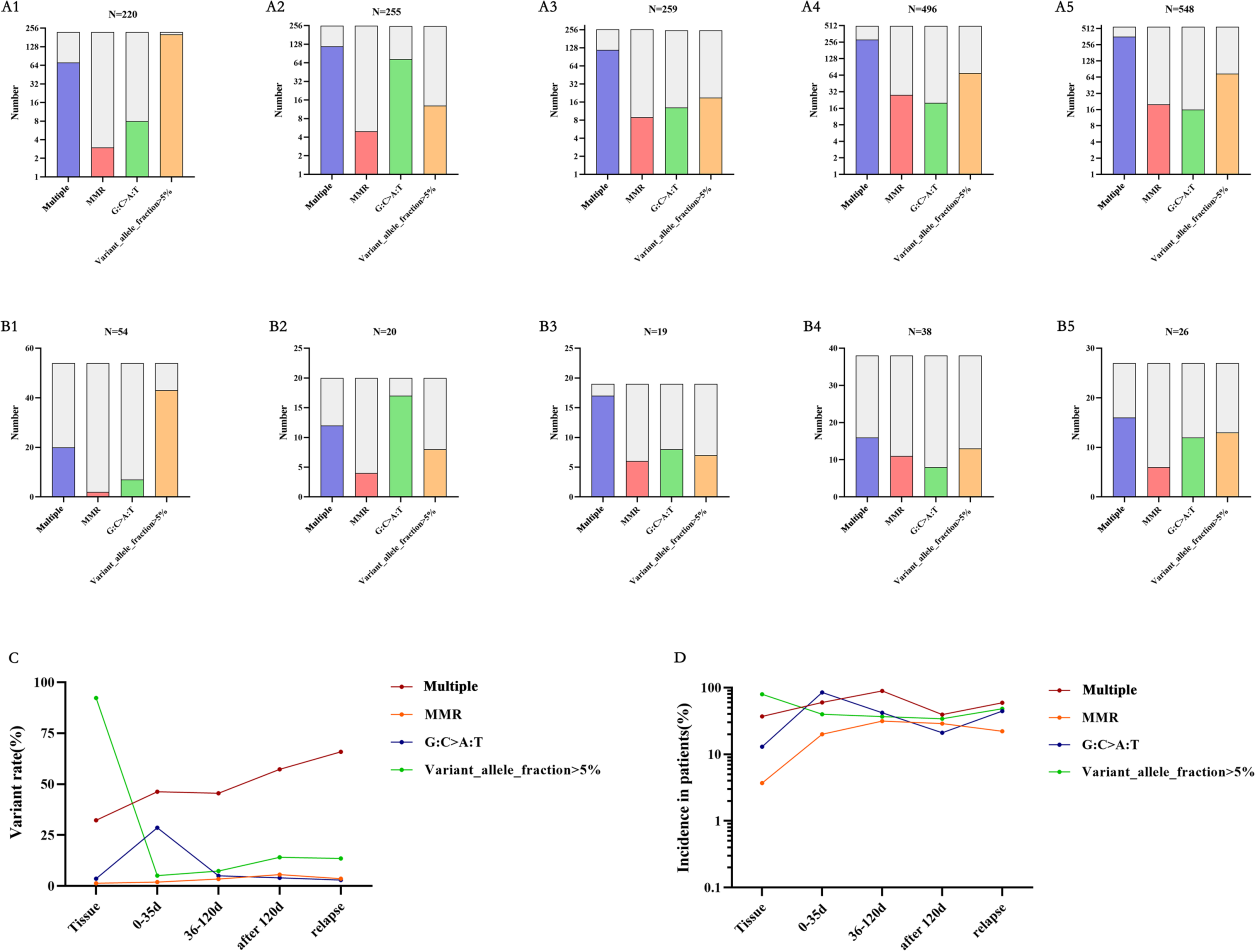
Mutations at different time points – multiple mutations, MMR-related mutations, temozolomide-related mutations, and VAF greater than 5%. **(A, B)** A1-A5 is from the data of each mutation site, and B1-B5 is from the data of each mutation patient. corresponding to the primary tumor tissue specimens, TISF specimens within 0-35 days after surgery, TISF specimens 36-120 days after surgery, TISF specimens 120 days after surgery, and TISF specimens of recurrent tumors. **(C, D)** Multiple mutations and MMR increased with tumor recurrence. VAF decreased significantly after tumor excision and increased with tumor recurrence.

In TISF-DNA, we found mismatch repair (MMR) genes (MSH2, MSH3, MSH6, MLH1, MLH3, PMS1, PMS2), and temozolomide-related high mutations [the accumulation of G: C>A: T: transitions at non-CPg sites in hypermutated gliomas after exposure to alkylating Agents (20, 21)]. In general, Multiple mutations, MMR-related mutation, and gene mutation with VAF > 5% increased gradually in gene mutation with postoperative treatment and tumor progression after glioma resection, and the proportion of patients also increased gradually (**Figures 4A1-5, B1-5**). All patients were treated with temozolomide (alkylating agent) chemotherapy after surgery. Interestingly, as chemotherapy progressed, we did not find an increase in the mutation frequency of temozolomide-related high mutations, nor did we find an increase in the number of patients with temozolomide-related high mutations (**Figure 4C, D**). Therefore, MMR and Multiple mutations may be associated with glioma recurrence after treatment, which is consistent with previous studies.

### Genomic characterization of glioma patients was continuously monitored

**Figure 5:**
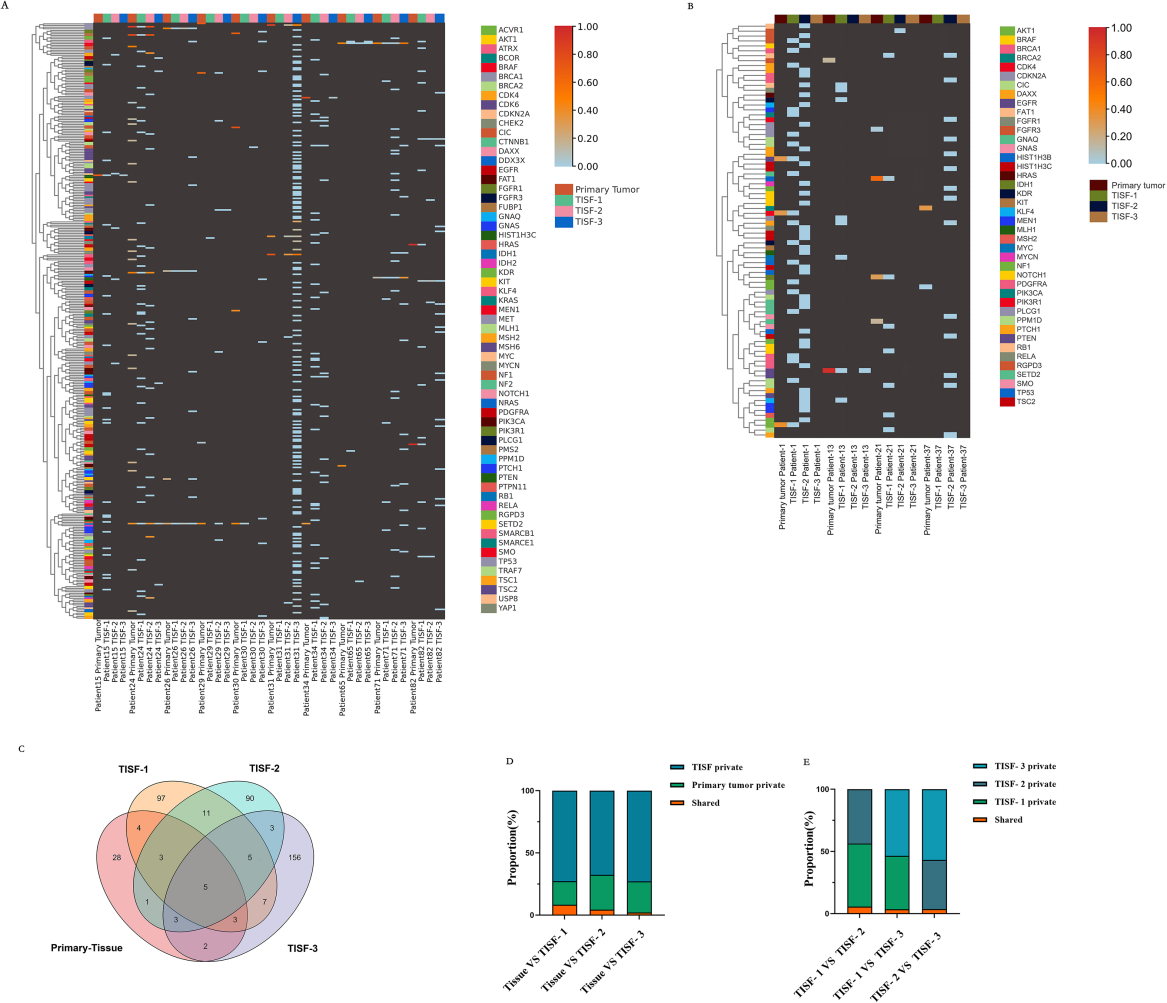
Characteristics of genetic mutations at different times in 14 patients from continuous TISF samples. **(A)** Genomic landscape of 10 recurrent patients at follow-up. Color heat represented VAF values, which were elevated at the mutated locus at recurrence. Hypermutation was found when patient NO.31 recurred. **(B)** Genomic landscape of mutations in 4 patients without recurrence at follow-up. The mutation locus VAF was not elevated at the time of recurrence. **(C)** There were differences in mutation sites at different periods in all recurrent patients during continuous testing. There are only five sites throughout the relapse process: IDH1p Arg132His, TP53p. Arg273Cys, PIK3Cap. Gln546Lys, HIST1H3Cp. Lys37Met, BRAFp. Val600Glu. TISF samples contain much more private mutations. **(D, E)** The proportion of shared mutations and private mutations among different test samples. TISF samples contained much more private mutations, and shared mutations accounted for only a small proportion, which was consistent with the previous results.

TISF samples from 14 patients in this study were obtained by continuous monitoring, TISF-1 (postoperative 35 days), TISF-2 (postoperative 36-120 days), and TISF-3 (postoperative 120 days), and they were divided into two groups: non-recurrence group (n = 4) and recurrence group (n = 10). Similar to the above conclusions, low-frequency distribution was observed in both groups at the early stage after glioma resection (**Figures 5A, B**). However, increased frequency of some genes in TISF-3 was observed in the recurrence group while not in the non-recurrence group. With the postoperative progression of glioma, there were more private mutations in TISF-DNA than in the primary tissue (**Figure 5C**), with a maximum of 156 mutations in TISF-3 Mutations shared between TISF and primary tissue decreased gradually, with the highest in TISF-1 and decreased with tumor progression (**Figure 5D**). A lower proportion of mutations shared between TISF-1 and TISF-3 was also found in TISF-DNA samples (**Figure 5E**). This suggests that the minimal residual disease in the early postoperative stage may be closer to the primary tumor; there is significant heterogeneity between recurrent and primary tumors.

**Figure 6:**
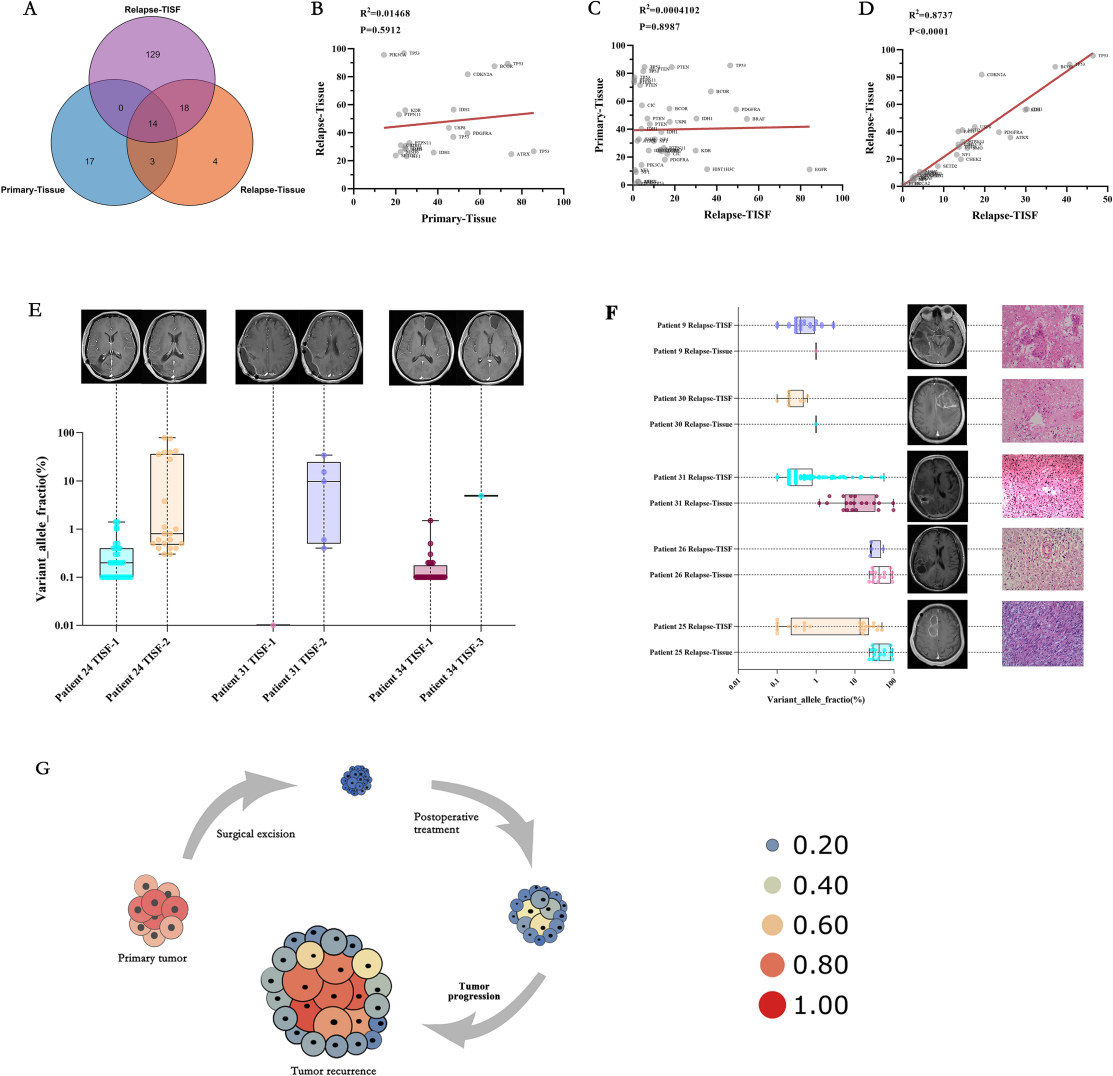
Characteristics and clinical relevance of VAF gene mutations among TISF samples. **(A)** There were many mutated sites in the TISF of recurrent tumors, including most of the recurrent tissues (81.8%) and only 8.6% identical to the primary tumor tissue. **(B)** In the five patients who underwent the second operation, there was no significant correlation between the shared mutant VAF between the primary tumor tissue and the recurrent tumor tissue, which reflected the differences in the tumor recurrence driver genes. **(C)** Similarly, no significant association was found between primary tumor tissue and recurrent tumor TISF samples for shared mutant VAF. **(D)** In the TISF samples of recurrent tumor tissue and recurrent tumor, the VAF of shared mutation was highly consistent and positively correlated. **(E)** Allele variation frequency increase in TISF was found in 3 patients with glioma after surgery before positive imaging examination, which may indicate recurrence of tumor DNA level. **(F)** Five patients underwent a second operation, and pathology confirmed pseudotumor progression in patients 9 and 30, with lower VAF values for the mutated gene in their relapsed tissues and relapsed TISF. Patients 31, 26, and 25 had pathologically confirmed tumor recurrence and had higher VAF values for the mutated gene in their recurrent tissues and recurrent TISF. **(G)** We derive the evolutionary cell model of recurrent glioma under the pressure of first-line therapy. Size and heat represent the VAF value of the mutant gene. With tumor recurrence, mutated genes gradually evolved from low-frequency VAF to dominant driver genes, and VAF gradually increased until tumor recurrence was found on imaging.

Five patients underwent a second surgical resection after recurrence, and 129 gene loci mutations were detected in the recurrent TISF but not in the recurrent tissue (**Figure 6A**), and only 32 loci were detected in the recurrent tissue. There were only 17 identical mutation loci in the primary and recurrent tumor, with a consistency rate of only 30.36%. There was no significant correlation between the frequency of mutations in the genes consistent with the primary and recurrent tissues (**Figure 6B**, P = 0.5912, R^2^ = 0.01468). There was no significant correlation between the frequency of mutations shared between TISF samples from 26 recurrent patients and their tissues (**Figure 6C**, P = 0.8987, R^2^ = 0.0004102). However, a positive correlation was found between the frequency of shared mutations in relapsed tissues and relapsed TISF (**Figure 6D**, P < 0.0001, R^2^ = 0.8737).

### Clinical relevance of genomic changes in glioma

Based on the results of this study, we summarized the correlation between genomic changes after glioma surgery and clinical practice. We found that the elevation of VAF predates radiographic findings (**Figure 6E**, Patient 24, patient 31, and patient 34), indicating that tumor DNA relapse may be present when radiographic findings are not positive and is an ultra-early manifestation of relapse. We performed a second tumor resection for six patients with radiological manifestations of recurrence, and only three patients were found to have pathological manifestations of tumor recurrence, with Multiple mutations and high VAF values in their recurrent TISF. Two patients presented with extensive inflammatory and necrotic tissue, and no mutations were detected in their TISF (**Figure 6F**). Finally, we summarize the evolutionary model of first-line therapy in glioma cases (**Figure 6G**), in which tumor-driving genes are removed along with surgical excision of the primary tumor. There are many low-frequency mutations in the residual disease stage after surgery. After the treatment, the VAF of mutation increased under the pressure of the treatment, at which time the imaging may not show a positive result. As the tumor evolves further, tumor recurrence driver genes emerge, leading to tumor growth and imaging recurrence. These results indicate that early molecular recurrence of glioma may be effectively detected by TISF-DNA, providing a possibility for early clinical diagnosis and treatment of recurrence.

## Discussion

Although tumor biopsy remains the gold standard for glioma diagnosis, liquid biopsy-based on TISF-DNA overcomes many of the limitations of tumor biopsy and has clinical advantages over circulating tumor DNA derived from blood and cerebrospinal fluid (19). The biopsy is sampled locally at a single metastatic site, which may introduce sampling bias (22). Biopsies can be painful and cause anxiety, and biopsies carry the risk of bleeding or infection. CSF-ctDNA is a suitable method, but it is almost difficult to detect in the early stage after tumor resection. Positive CSF-ctDNA often requires certain conditions, including large tumor load, tumor progression, and tumor diffusion into the ventricular system or subarachnoid space (14–16). This makes it impossible to detect genomic changes at the early stage of glioma evolution, let alone detect tumor recurrence at the tumor DNA level earlier than imaging, making early postoperative targeted therapy more difficult.

Our analysis of glioma driver gene heterogeneity and recurrence provides essential insights into the genomic state at all stages of glioma first-line treatment. First, in the first-line treatment context, the genetic mutations of the tumor genome differ at different stages (**Figure 2A-D**). In the early stage after surgical resection, the gene status was dominated by low-VAF mutations, and no high-VAF mutations appeared at this time. As the treatment progresses, tumor Multiple mutations increase, and VAF increase (**Figure 4C, D**). In addition, we observed that mutations detected in recurrent tumor tissue samples were in good agreement with matched TISF samples (**Figure 6D**), demonstrating the reliability of TISF-DNA in detecting tumor DNA variation during systemic treatment of gliomas.

Our study also found that TISF-DNA found increased tumor DNA VAF levels, but there was no positive imaging performance at this time (**Figure 6E**). At this time, the tumor had DNA level recurrence, which relevant studies have confirmed, and we found this for the first time in glioma (10, 23–25). In addition, the TISF of patients with recurrence showed high-frequency mutations similar to that of the recurrent tumor tissue. In contrast, the TISF of patients with pseudoprogression showed low-frequency mutations, and tissue test was negative, but they all showed positive findings on imaging. This indicates that TISF-DNA can identify pseudoprogression of glioma in clinical practice, thus providing therapeutic guidance for physicians. Nevertheless, no similar study has been done.

## Conclusions

Our results show the state of the genome and the course of relapse at different stages under pressure from the first-line treatment of glioma. We determined that continuous TISF can be used to track postoperative residual disease, tumor relapse, and pseudoprogression of gliomas. It can dynamically provide guidance for the clinical management of glioma patients, promote the development of glioma-related research.

## Data Availability

The original contributions presented in the study are publicly available. This data can be found here: [https://figshare.com/articles/dataset/Data/19203251?file=34114886].
